# Iron-sulfur clusters: the road to room temperature

**DOI:** 10.1007/s00775-025-02094-0

**Published:** 2025-01-31

**Authors:** Brighton A. Skeel, Daniel L. M. Suess

**Affiliations:** Department of Chemistry, Massachusetts Institute of Technology, Cambridge, MA USA

**Keywords:** Iron-sulfur proteins, Metalloenzymes, Electronic structure, Exchange coupling, Clusters

## Abstract

Iron-sulfur proteins perform a wide variety of reactions central to the metabolisms of all living organisms. Foundational to their reaction chemistry are the rich electronic structures of their constituent Fe-S clusters, which differ in important ways from the active sites of mononuclear Fe enzymes. In this perspective, we summarize the essential electronic structure features that make Fe-S clusters unique, and point to the need for studies aimed at understanding the electronic basis for their reactivity under physiological conditions. Specifically, at ambient temperature, both the ground state and a large number of excited states are thermally populated, and thus a complete understanding of Fe-S cluster reactivity must take into account the properties, energies, and reactivity patterns of these excited states. We highlight prior research toward characterizing the low-energy excited states of Fe-S clusters that has established what is now a consensus model of these excited state manifolds and the bonding interactions that give rise to them. In particular, we discuss the low-energy alternate spin states and valence electron configurations that occur in Fe-S clusters of varying nuclearities, and finally suggest that there may be unrecognized functional roles for these states.

**Graphical abstract:**

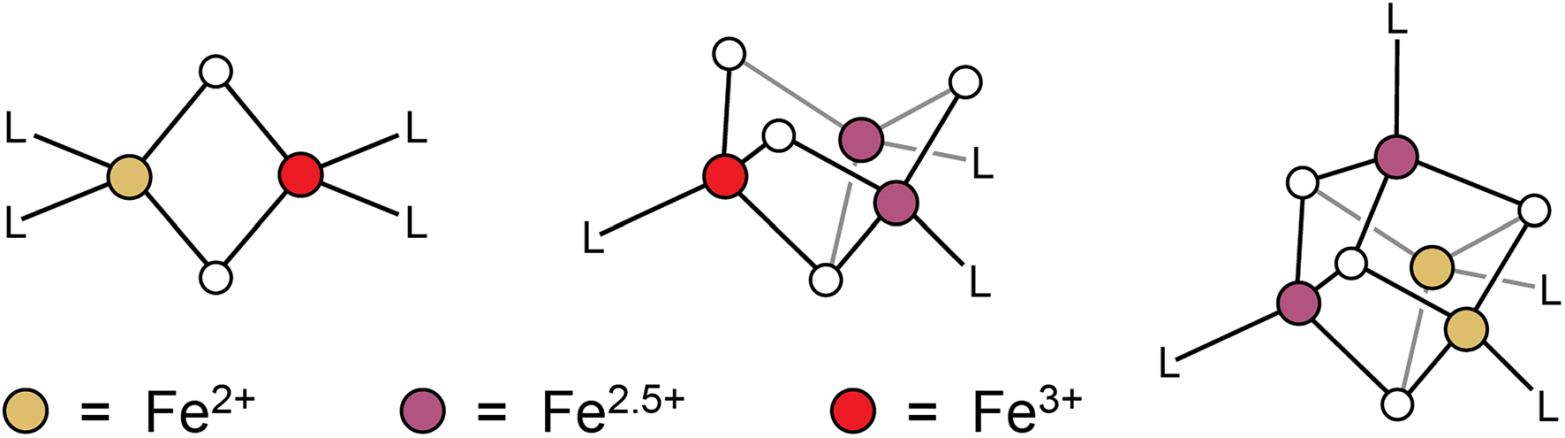

## Introduction

It is becoming increasingly recognized that studies of biomolecules must focus on understanding or otherwise rationalizing their reactivity under the conditions in which they perform their native functions. For Fe-S proteins in particular, doing so requires contending with the rich electronic structures of Fe-S clusters at physiological temperatures, which remains a formidable task for both theorists and experimentalists. Research thrusts in this area have been and will continue to be guided by past work that has yielded the contemporary picture of Fe-S cluster electronic structure, and it is therefore useful to understand the historic arc of research as it has led to this point. We sketch some essential aspects of this history here.

The story begins in 1960 with Beinert’s observation of a new kind of signal in the EPR spectra of certain reduced, non-heme Fe-containing enzymes and enzymatic preparations [1, 2]. In the next few years, a number of these enzymes, some of which had been given the classifier “ferredoxins,” were identified, and interest in their chemical composition had developed. In 1963, Rabinowitz described the labile Fe content, as well as the presence of inorganic sulfide and its content, in the clostridial ferredoxins [3, 4]. It was noted that, oddly, there was a significant quantity of Fe^2+^ consistently observed, in spite of the fact that the protein was isolated in what was nominally known to be the oxidized state. Between this point and 1972, a series of publications describing in more detail the electron paramagnetic resonance (EPR) [5, 6], Mössbauer [7], and nuclear magnetic resonance (NMR) [8] spectra of the clostridial ferredoxins appeared, each contributing to the growing set of constraints the structure of the Fe-containing portion of the protein must have satisfied. At this point, speculation as to the structure of the Fe-S cofactors in these proteins began to appear [7, 8], and although it was ultimately recognized that these early proposed structures were incorrect, many of the essential features predicted (*i.e.,* the presence of Fe-cysteine bonds, bridging inorganic sulfides, and tetrahedral Fe sites) were accurate.

Simultaneously, work was being carried out on a more diverse set of Fe-S proteins. In 1966, a pioneering interpretation of the EPR spectrum of the reduced spinach ferredoxin (now known to be an [Fe_2_S_2_]^1+^ cluster) was published by Gibson [9]. This work successfully rationalized the data in terms of the ferredoxin’s metallocofactor being composed of a pair of antiferromagnetically coupled, high-spin Fe^2+^ and Fe^3+^ ions; it was one of the first successful attempts at understanding the electronic structure of a ferredoxin, and is a standard model to this day. By this point, other classes of Fe-S proteins had also been identified. Of note, several so-called “high-potential iron proteins” (HiPIPs) from different organisms, distinguished by their significantly higher midpoint redox potentials relative to the ferredoxins, had been characterized in terms of their Fe and S content by 1967 [10], and by low-resolution X-ray diffraction in 1968 [11]. The latter indicated that all four Fe ions contained by these HiPIPs were present in a single cluster, though further structural information regarding the nature of this cluster was elusive.

Finally, in 1972, an X-ray crystal structure of the *C. pasteurianum* ferredoxin was published [12]. The resolution of the structure was sufficient to observe what was modeled as a pair of cuboidal, [Fe_4_S_4_] clusters, each bound by four cysteine thiolates—a structural motif strikingly similar to the cysteine thiolate-ligated [Fe_4_S_4_] cluster that had been reported within the *C. vinosum* HiPIP the same year, also obtained by X-ray diffraction [13]—suggesting the inorganic Fe-S cluster motif may be common to multiple protein families. Trailing this pair of results by mere months, Holm published the first high-resolution structure of a cuboidal [Fe_4_S_4_] cluster with the correct tetrahedral ligation of the Fe sites: [Et_4_N]_2_[Fe_4_S_4_(SBn)_4_] (Bn = benzyl; Et = ethyl) [14]. The tetrahedral coordination of the Fe sites, and the valences of the constituent Fe ions (two Fe^2+^ and two Fe^3+^ ions, formally), distinguished this work from previous, related work by Dahl, who had reported the preparation of Cp_4_Fe_4_S_4_ (Cp = cyclopentadienyl), an all-ferric [Fe_4_S_4_] cluster containing locally pseudo-octahedral Fe sites [15].

Holm’s publication of this structure can be viewed as a turning point for the field of Fe-S cluster chemistry and in synthetic modeling chemistry more generally. From here onward, a sustained collaboration between biologists, chemists, physicists, and theorists developed, culminating in what has become the interdisciplinary area of study that exists today. Some of the critical experimental and theoretical results that have emerged from these research thrusts and that lie on “the road to room temperature” are summarized in the following sections.

## Essential aspects of Fe-S cluster electronic structure

### Valences, antiferromagnetic coupling, and spin-dependent electron delocalization

A description of Fe-S clusters begins with their compositions: high-spin tetrahedral Fe^2+^ and Fe^3+^ ions, and bridging inorganic sulfide ions, S^2−^ [16]. These restrictions encompass the great majority of Fe-S clusters, with some exceptions related to the valence distribution [17] and to the coordination number (*e.g*., those with higher-coordinate Fe sites, such as is found in members of the radical *S*-adenosylmethonine superfamily [18, 19], as well as rare examples of clusters featuring lower-coordinate Fe sites [20, 21]). This definition also intentionally excludes certain compounds (such as the example given previously: Cp_4_Fe_4_S_4_) [15] whose electronic properties are distinct from known biological Fe-S clusters.

**Fig. 1:**
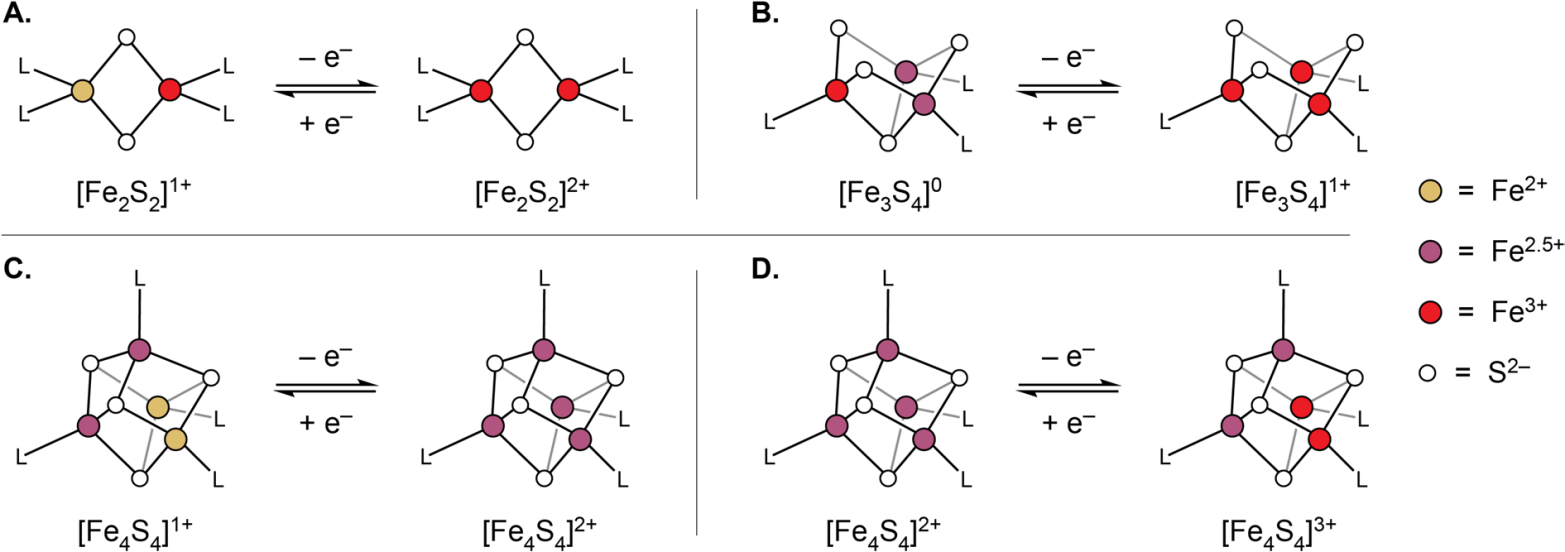
Most frequently encountered biological Fe-S cluster motifs. **A** [Fe_2_S_2_] clusters. **B** [Fe_3_S_4_] clusters. **C** [Fe_4_S_4_] clusters in ferredoxins. **D** [Fe_4_S_4_] clusters in HiPIPs

In biological systems, Fe-S clusters are found in protein scaffolds and occur in varying nuclearities and topologies, with the most common motifs being [Fe_2_S_2_]^1+/2+^ clusters, open-cuboidal [Fe_3_S_4_]^0/1+^ clusters, and cuboidal [Fe_4_S_4_]^1+/2+^ or [Fe_4_S_4_]^2+/3+^ clusters (*e.g*., in the previously discussed ferredoxins and HiPIPs, respectively) (Fig. 1). Although the characterization of reaction intermediates and models thereof [22] continues to expand the number and types of ligands bound to Fe-S clusters, we focus here on those found in the resting-state structures. In such structures, cysteine thiolates are the most common terminal ligands, but alternative amino acids [23] such as aspartate, glutamate, histidine, serine [24], tyrosine [25], arginine [26], threonine [27], and methionine [28], as well as solvent and small molecules, have been shown to bind to one or, in certain [Fe_2_S_2_] systems [29–31], two Fe sites. The most common alternative, protein-derived ligands are carboxylates or histidine residues, with the remaining amino acids listed previously being considerably rarer.

**Fig. 2:**
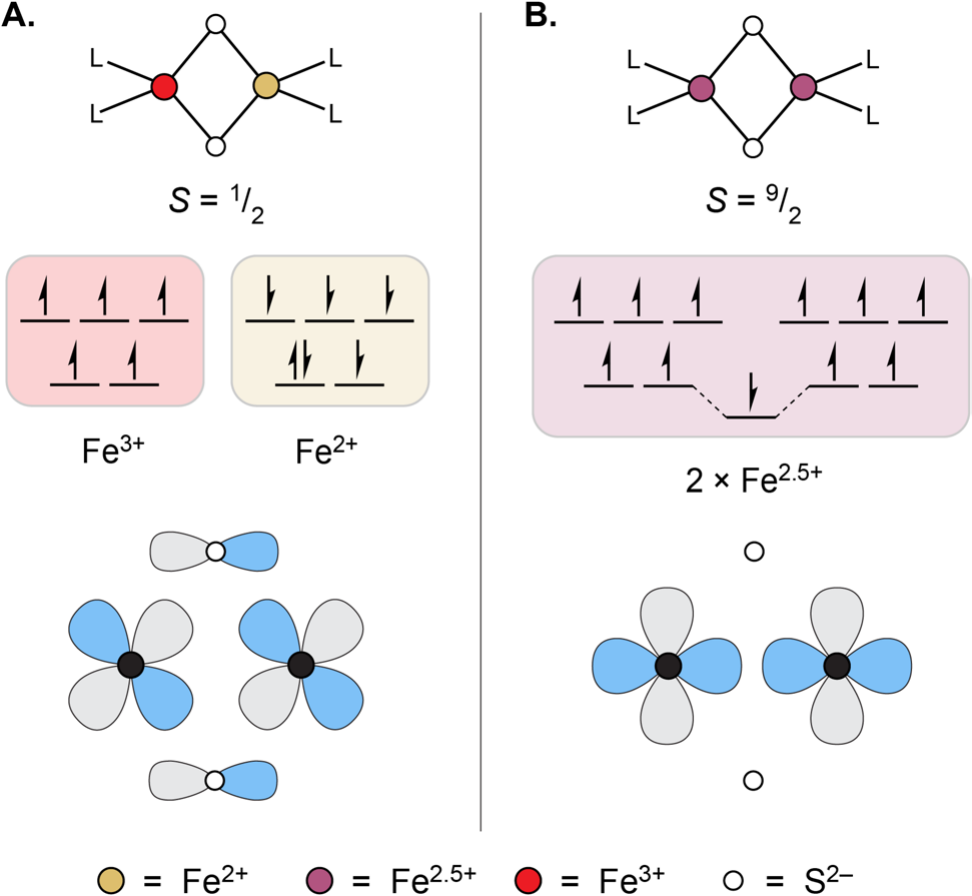
Two limiting valence and spin patterns for a representative [Fe_2_S_2_]^1+^ cluster (upper) with the orbital interactions that favor either configuration (lower). **A** Localized valences and spin anti-alignment favored by sulfide-mediated antiferromagnetic superexchange. **B** Mixed valency and spin alignment favored by double exchange. Note that the orbital interactions depicted in both panels are examples of multiple possible exchange pathways, of which several generally exist

Two spin coupling mechanisms are ubiquitous in Fe-S cluster chemistry: bridging sulfide-mediated superexchange coupling (Fig. 2) and electron hopping between Fe sites, in particular those of differing valence (pairs composed of an Fe^2+^ and Fe^3+^ ion, specifically), which is termed spin-dependent electron delocalization (or “double-exchange”; Fig. 2).

Superexchange interactions are generally described in the Fe-S cluster literature using the Heisenberg Hamiltonian, which gives the energy of a given total spin state $S$ as a function of the coupling $J$ between the system’s constituent spins, taken as the vector product between them. The prototypical effective Hamiltonian, defined for a two-spin system with $\overrightarrow{S}={\overrightarrow{S}}_{1}+{\overrightarrow{S}}_{2}$, is given by:

1 $${\widehat{H}}_{Heis}=J{\overrightarrow{S}}_{1}\cdot {\overrightarrow{S}}_{2}$$

Here, we take the convention where the factor multiplying ${\overrightarrow{S}}_{1}\cdot {\overrightarrow{S}}_{2}$ is given as $J$, but note that this factor can also be given as $-2J$ or $-J$. For this casting of the Heisenberg Hamiltonian, the relative energies of the spin states are given by

2 $$E=\frac{J}{2}S(S+1)$$

and the values of $S$ are given in terms of ${S}_{1}$ and ${S}_{2}$ by the triangle inequality

3 $$|{S}_{1}-{S}_{2}|\le S\le |{S}_{1}+{S}_{2}|$$

It is then the case that antiferromagnetic coupling, which favors low overall spin states and spin anti-alignment between sites, occurs when $J$ is positive, and ferromagnetic coupling, which favors high spin states and spin alignment between sites, occurs when $J$ is negative.

Discussing cuboidal [Fe_4_S_4_]^n+^ clusters as a representative system, it is evident that the spin–spin interactions within Fe-S clusters are phenomenologically antiferromagnetic in nature when considered as a whole. In particular, the overwhelming majority of clusters in their biologically relevant core charge states (*n* = 0, 1, 2, or 3) are either $S=0$ ([Fe_4_S_4_]^2+^ clusters) or $S=1/2$ ([Fe_4_S_4_]^1+/3+^ clusters) in their ground states—the lowest possible spins attainable by coupling together pairs of high spin Fe^2+^ (individually $S=2$) and high spin Fe^3+^ (individually $S=5/2$) [32–34]. Correspondingly, values for the superexchange coupling constant $J$ in Fe-S clusters (where they have been measured) are positive, and thus antiferromagnetic [32, 33, 35–37].

Double exchange is a more specific interaction within Fe-S clusters in that, rather than acting between each pair of Fe ions, it is typically only appreciably operative within heterovalent pairs of Fe sites: namely, spin-aligned pairs of Fe^2+^ and Fe^3+^. This phenomenon may be described by considering a pair of spin-aligned Fe^3+^ ions and adding an electron to the system (or, equivalently, a pair of spin-aligned Fe^2+^ ions and removing one electron). In this case, while it is possible to add the electron entirely to one Fe center, giving an Fe^2+^ and an Fe^3+^ ion, it may be more favorable for the excess electron to be delocalized over the two sites, giving rise to a pair of mixed-valence Fe^2.5+^ ions. The extent to which this delocalized state is favored over the valence-trapped state is proportional to the extent to which the individual spin centers are coaligned, with parallel alignment being the most favorable, and antiparallel alignment being the least. That the extent of this stabilization is dependent on the degree of site spin collinearity arises from favorable exchange interactions between the itinerant electron and the static electrons on each Fe site, which are maximized when the spin moments on each site are aligned. Considering double exchange as an interaction between two metal-centered orbitals, this interaction gives rise to an in-phase “bonding” and an out of phase “anti-bonding” set of orbitals (such nomenclature being used only loosely), with the energy of either state given by [38]:

4 $${E}_{double exchange}=\pm B\left(S+\frac{1}{2}\right)$$

where the value *B* is the double exchange constant. Thus, for larger values of $S$, the double exchange interaction is more stabilizing.

The need for this term in describing the electronic structures of Fe-S clusters arose over time as mounting evidence suggested that discreet pairs of Fe^2+^/Fe^3+^ ions were not present in clusters with more than two Fe ions, but rather that these Fe ions were equivalent (by, for instance, Mössbauer spectroscopy) in a pairwise fashion [36, 39–41]. On all timescales measured so far [42], this electron delocalization, as it exists in [Fe_4_S_4_] clusters, may be considered class III within the Robin-Day classification scheme [43]. This is not true of all cluster topologies, with [Fe_2_S_2_]^1+^ clusters being the notable exceptions (though examples with extensive electron delocalization have been reported [44–46]). The tendency toward valence localization in dinuclear systems occurs because the stabilization brought about by double exchange is typically less than the energy required to defeat the antiferromagnetic superexchange interactions between the two Fe centers, so these particular systems are most frequently valence-trapped (note that vibronic coupling effects also play an important role in favoring valence trapped configurations [46]).

It is then the confluence of superexchange and double exchange interactions that produce the ground states of Fe-S clusters. These exchange coupling models and their associated Hamiltonians have been extensively discussed elsewhere [33, 39, 41, 47], and it is more useful for our purposes to sketch a simple, qualitative picture of their essential features, here for the specific case of the ubiquitous cuboidal [Fe_4_S_4_] cluster topology. When discussing the electronic structure picture of a cuboidal [Fe_4_S_4_] cluster, one most commonly works within the so-called “pair-of-pairs” framework (the Heisenberg double exchange (“HDE”) Hamiltonian), in which the good quantum numbers describing the system are:

- the site spins, ${S}_{1}$, ${S}_{2}$, ${S}_{3}$, and ${S}_{4}$, each describing the spin quantum number of one Fe site, numbered 1–4,
- the spins of two pairs of Fe ions, ${S}_{12}$ and ${S}_{34}$, corresponding to the spin quantum numbers of the pairs defined as Fe1-Fe2 and Fe3-Fe4, and
- the total spin of the cluster, $S$.

The quantum numbers listed above are good quantum numbers in the pair-of-pairs model on account of symmetry constraints imposed on the nature of the intracluster Fe–Fe interactions. In the general case, where all Fe–Fe interactions may differ, only the site spins and total spin are required to be good quantum numbers. This model Hamiltonian has several appealing properties, two important ones being that it is sufficiently complex to capture much of the essential physics of [Fe_4_S_4_] systems, and that it has chemically meaningful analytic solutions. Further, as this Hamiltonian retains two good intermediate spin quantum numbers, any given cluster spin state may be succinctly described using the notation $|{S}_{1}\,\,{ S}_{2}\,\, {S}_{3} \,\,{S}_{4} \,\,{S}_{12}\,\, {S}_{34}\,\, S\rangle$, which is frequently shortened to the $|{S}_{12}\,\,{S}_{34}\,\,S\rangle$, where the spins of the individual sites are implied.

**Fig. 3:**
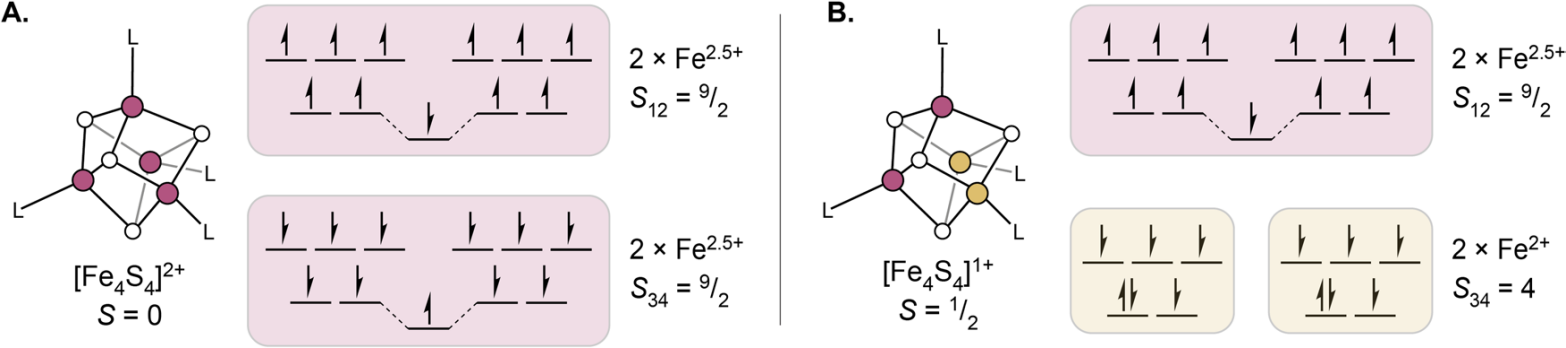
Ground state spin and valence pictures for some common [Fe_4_S_4_] charge states. **A** Depiction of the $|9/2\,\,9/2\,\,0\rangle$ spin state for the [Fe_4_S_4_]^2+^ cluster charge state. **B** Depiction of the $|4\,\,9/2\,\,1/2\rangle$ spin state for the reduced, [Fe_4_S_4_]^1+^ cluster charge state

The ground state for [Fe_4_S_4_]^2+^ clusters in this notation is, with exceedingly rare exceptions [48], $|9/2\,\, 9/2\,\, 0\rangle$, which is to say the cluster is composed of two magnetically equivalent, spin-aligned pairs of high-spin Fe^2+^ and Fe^3+^ ions (Fig. 3A). Within these spin-aligned pairs, the double exchange interaction is strong, and the valences are best described as Fe^2.5+^, so overall the cluster is composed of 2 × 2 × Fe^2.5+^ ions. While the spin alignment that occurs within each of these pairs may seem unfavorable in light of the antiferromagnetic coupling between individual sites, it is favorable in the global sense that this configuration satisfies four other antiferromagnetic interactions between Fe sites in different pairs. The additional factor of double exchange stabilizes this particular ground state further.

In the reduced, [Fe_4_S_4_]^1+^ state, the typical ground state is given by $|4\,\, 9/2\,\, 1/2\rangle$, where again there is a mixed-valence pair of Fe ions (sites 3 and 4), but now also a pair of spin-aligned ferrous ions (sites 1 and 2) between which there is no meaningful double-exchange interaction (Fig. 3B). Thus, the valence picture of these clusters is given as 2 × Fe^2+^ and 2 × Fe^2.5+^. A similar valence picture holds for the oxidized [Fe_4_S_4_]^3+^ state, where there are 2 × Fe^3+^ and 2 × Fe^2.5+^ ions, with the caveat that rather than adopting maximal alignment of spins within each pair of Fe ions (which would be the $|5\,\,9/2 \,\,1/2\rangle$ state for sites 1 and 2 being the ferric ions and sites 3 and 4 being the mixed-valent ions), some degree of spin canting occurs in the ground states of these clusters, which have been described as either $|4\,\, 9/2 \,\,1/2\rangle$ or $|3\,\, 7/2\,\, 1/2\rangle$ [32, 49]. (Note that such states involving spin canting are not so straightforwardly depicted using the kinds of spin vector representations invoked in Figs. 2 and 3.) The most relevant feature here is that the magnitude of the spin for the ferric pair is smaller than that of the mixed-valence pair, an observation first noted when the pair-specific ^57^Fe hyperfine tensors determined by magnetic Mössbauer spectroscopy of the oxidized clusters were reported [32, 50].

### The low-energy excited states of Fe-S clusters

**Fig. 4:**
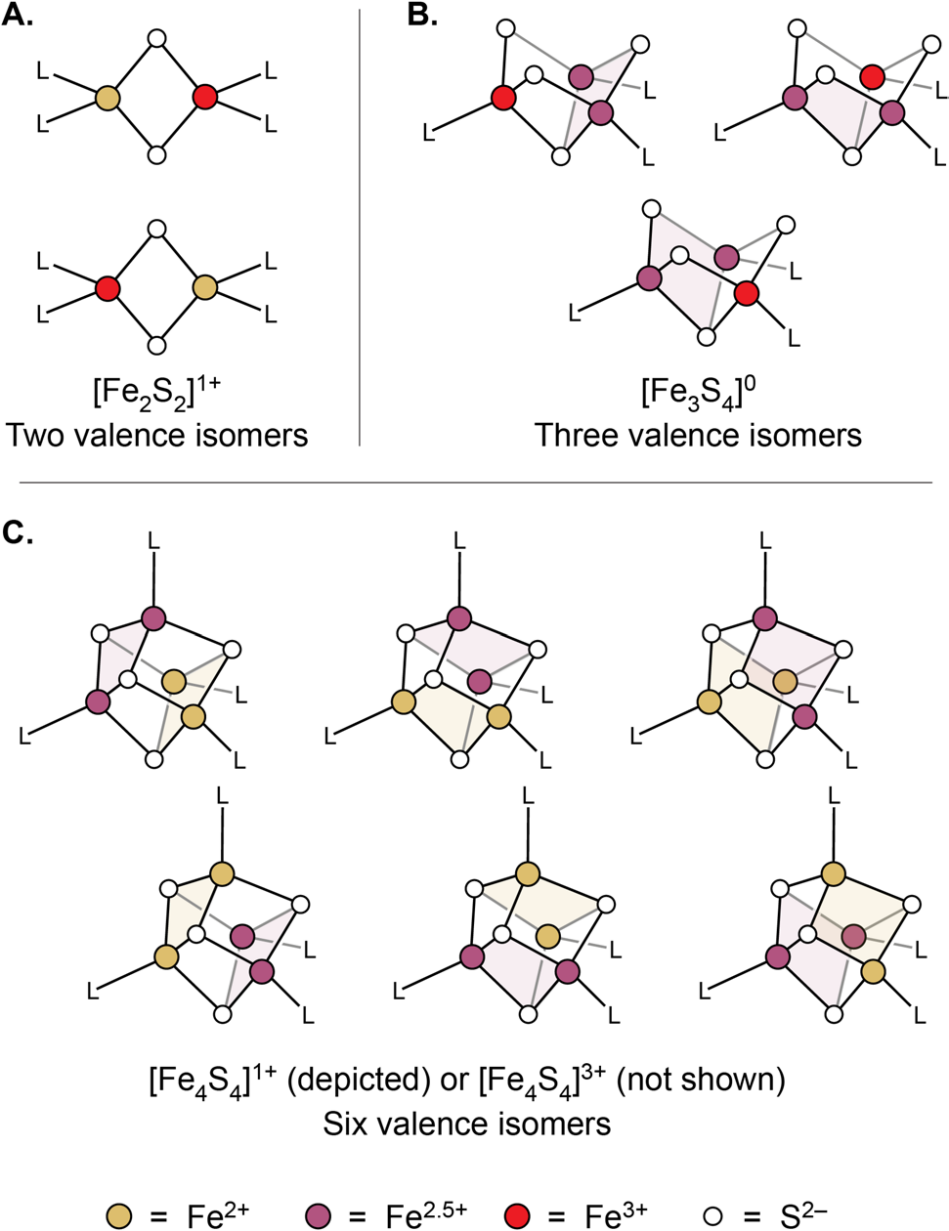
Valence isomer states for different Fe-S cluster topologies. **A** Two valence localized valence isomer states for an [Fe_2_S_2_]^1+^ cluster. **B** Three valence isomer states for an [Fe_3_S_4_]^0^ cluster. **C** Six valence isomer states for an [Fe_4_S_4_]^1+^ cluster; note that [Fe_4_S_4_]^3+^ clusters retain the same pattern of valence isomer states but with Fe^3+^ ions in place of Fe^2+^ ions. Shaded rhombs indicate spin-aligned pairs of Fe centers

The previous section dealt entirely with models describing the ground states of Fe-S clusters. Determining the ground state picture for these systems has been one of the great successes of the field, but it must be pointed out that for a [Fe_4_S_4_]^1+^ cluster, even the simple HDE Hamiltonian produces a total of 180 different spin states, many of which are low in energy. Further, computational work suggests this is a gross underestimate of the density of excited states in these systems [51]. The thermal population of excited states is easily inferred by, for instance, considering the room temperature magnetic moments of [Fe_4_S_4_]^1+^ clusters, which typically span a range between 3 and 4.5 Bohr magnetons [33, 52–55]—far higher than the spin-only value expected for the ground state spin of $S=1/2$ (1.73 Bohr magnetons). This implies the very significant population of excited states with total spin greater than $S=1/2$, and that a significant number of the 179 excited states predicted by the HDE model must be, in fact, thermally accessible. Complicating matters further, in Fe-S clusters containing more than one formal Fe valence (*e.g.*, the Fe^2+^ and Fe^2.5+^ in [Fe_4_S_4_]^1+^ clusters), there exist multiple spatial arrangements of the valences (“valence isomers,” also called “electronic isomers” or “electromers;” Fig. 4) each of which is associated with its own spin ladder. The identity of the ground state valence electron distribution, and how well isolated this particular configuration is, has been discussed for a variety of cluster topologies, including the [Fe_2_S_2_]^1+^, [Fe_3_S_4_]^0^, and [Fe_4_S_4_]^1+/3+^ states [56–60], and we have recently quantitatively evaluated the energetic separation of valence isomers in [Fe_4_S_4_]^1+^ clusters and determined its dependency on the identity of a cluster’s ligands [37].

This measurable depopulation of the ground state at and around room-temperature is profoundly important (and presumably even more so for hyperthermophilic organisms), and brings with it the possibility that the excited states of these Fe-S cluster systems may directly bear on their room-temperature reactivity properties. The topic of excited states is also one of historic importance, and there has been, in general, a great deal of interest in describing the nature and energies of Fe-S cluster excited states. These lines of inquiry can be classified as either experimental or theoretical in nature, which we discuss separately below.

Experimental work describing the excited states of Fe-S clusters generally takes one of two approaches. The first approach entails imposing a model exchange coupling Hamiltonian on the system, fitting the relevant Hamiltonian parameters, and then computing the energies and properties of the excited states as given by the spin eigenfunctions and associated energies of the model Hamiltonian (*i.e.*, a model-based approach). The second approach supposes the presence of one or more excited states and assumes some aspect of their properties (*e.g.*, the multiplicity of the excited state, its local spin projections, etc.) as relevant to the method of measurement employed, and then attempts to determine the energies of these states (*i.e.*, a model-agnostic approach). The first approach has the advantage of predicting more complete sets of states by extrapolating from the physics known to dominate in the ground state, but has the disadvantage that these models (*e.g*., the HDE Hamiltonian) may not fully describe the system. The second approach has the advantage of not being reliant upon an exchange coupling model that may or may not be suitable, with the significant drawback that only a limited number of states may be practically considered.

Speaking now specifically of studies on [Fe_4_S_4_] systems, quantitative model-based approaches have mostly centered on the use of magnetometry data as the observable [33, 35], sometimes supplemented with variable-temperature (VT) near-infrared absorption spectroscopy data [61]. High-symmetry systems have also been studied quantitatively using VT solid-state NMR spectroscopy [62], and work in our own lab has extended these ideas to lower symmetry systems by considering magnetometry and VT NMR data simultaneously [37]. This approach has opened the door to quantifying the energies—and thus the populations—of alternate spin and valence isomer states at room temperature. These quantitative studies have, to date, universally been conducted using synthetic [Fe_4_S_4_] compounds. Related descriptions of the excited state properties of Fe-S proteins, particularly as applied to the valence isomer problem, have made abundant use of both solution VT NMR and frozen-solution EPR spectroscopy [58, 63–65].

Model-independent approaches to describing the excited states of Fe-S clusters are, in general, less commonly employed, but have been applied to both synthetic [33, 35] and biological [66] systems, making use of either magnetometry data or variable temperature EPR. Magnetometry, in this application, has been used to identify the presence of several different spin states and their energies, whereas EPR spectroscopy has been used to identify the energy of a single excited state. The latter relies on assumptions about the dominant mechanism for electron spin relaxation in the system at hand, namely that the Orbach mechanism is operative.

Beyond experimental methods, the electronic structures of Fe-S clusters have attracted a great deal of attention from theorists, and have been the subject of many computational investigations. Early work in the area made use of either unrestricted Hartree–Fock or *X*α methods [67, 68]. The latter was an early variant of what soon became known as modern broken-symmetry density functional theory (BS-DFT) [69], which is the most common tool employed for computationally describing the electronic structures of Fe-S clusters at present [70]. While single-determinant BS-DFT is useful in general for studying Fe-S clusters, it is ultimately incapable of describing the multiconfigurational electronic structures of these systems, and in recent years there has been increasing impetus for the application of post-wavefunction methods to better understand Fe-S cluster properties. A number of these studies have appeared [51, 71–73] and, while all are promising, it remains the case (at least for the time being) that these methods are often too computationally expensive for widespread deployment, particularly when considering Fe-S clusters of higher nuclearity [73]. Common to all of these computational methods is an ability to predict (with varying degrees of granularity) a great deal of information pertaining to the properties of Fe-S cluster excited states. Of particular importance, computational methods may generally be used to compute parameters that appear in familiar experimental models, such as superexchange coupling values.

## Looking forward: functional roles for excited states?

The large number of thermally sampled, low-energy excited states in Fe-S clusters makes it particularly enticing to consider what role such excited states may play in dictating the reactivities of these metallocofactors. To date, much of the work toward this end has remained within the purview of theory, as reliable experimental pictures of the excited states of Fe-S clusters are sparse and not generally available for protein systems where reactivity patterns are of greatest interest. Early theory work in this area considered the possibility of excited-state regulation of electron transfer reactions, particularly in context of the spin states of these excited states [74, 75]. More recently, studies [76–78] employing density functional theory have examined the role of valence and spin distribution in controlling radical chemistry in radical *S*-adenosylmethionine catalysis, particularly with respect to the generation and reactivity of organometallic intermediates [79, 80]. As our collective understanding of the excited-state landscapes of Fe-S clusters expands, we anticipate that new links between electronic structure and reactivity will be made. These developments will require studies that experimentally link low-temperature electronic structure information to electronic structure attributes at ambient temperature. The road to room temperature is clear, and we now need only the studies to drive us there.

## Data Availability

No datasets were generated or analysed during the current study.

## Abbreviations

Bn: Benzyl
BS-DFT: Broken-symmetry density functional theory
Et: Ethyl
Cp: Cyclopentadienyl
EPR: Electron paramagnetic resonance
HDE: Heisenberg double exchange
HiPIP: High-potential iron protein
NMR: Nuclear magnetic resonance
VT: Variable-temperature
